# Expression and Characterization of an Alginate Lyase and Its Thermostable Mutant in *Pichia pastoris*

**DOI:** 10.3390/md18060305

**Published:** 2020-06-11

**Authors:** Suxiao Yang, Zhemin Liu, Xiaodan Fu, Changliang Zhu, Qing Kong, Min Yang, Haijin Mou

**Affiliations:** 1 College of Food Science and Engineering, Ocean University of China, Qingdao 266003, China; yangsuxiao66@163.com (S.Y.); ocean2013@126.com (Z.L.); luna_9303@163.com (X.F.); chlzhu@163.com (C.Z.); kongqing@ouc.edu.cn (Q.K.); 2 Yellow Sea Fisheries Research Institute, Chinese Academy of Fishery Sciences, Laboratory for Marine Drugs and Bioproducts of Pilot National laboratory for Marine Science and Technology, Qingdao 266071, China

**Keywords:** *Pichia pastoris*, alginate lyase, thermostable mutant, alginate oligosaccharides

## Abstract

Alginate is one of the most abundant polysaccharides in algae. Alginate lyase degrades alginate through a β-elimination mechanism to produce alginate oligosaccharides with special bioactivities. Improving enzyme activity and thermal stability can promote the application of alginate lyase in the industrial preparation of alginate oligosaccharides. In this study, the recombinant alginate lyase cAlyM and its thermostable mutant 102C300C were expressed and characterized in *Pichia pastoris*. The specific activities of cAlyM and 102C300C were 277.1 U/mg and 249.6 U/mg, respectively. Both enzymes showed maximal activity at 50 °C and pH 8.0 and polyG preference. The half-life values of 102C300C at 45 °C and 50 °C were 2.6 times and 11.7 times the values of cAlyM, respectively. The degradation products of 102C300C with a lower degree of polymerization contained more guluronate. The oligosaccharides with a polymerization degree of 2–4 were the final hydrolytic products. Therefore, 102C300C is potentially valuable in the production of alginate oligosaccharides with specific M/G ratio and molecular weights.

## 1. Introduction

Alginate is a natural anionic polysaccharide and one of the main structural components of brown algae, accounting for approximately 40% of the algal dry weight [1]. Structurally, alginate is an unbranched binary copolymer comprised of β-D-mannuronate (M) and α-L-guluronate (G) units. These units appear in three types of blocks: poly β-D-mannuronate (polyM), poly α-L-guluronate (polyG), and a heteropolymer (polyMG). As an abundant marine biomass and inexpensive material, alginate has been used in many fields, predominantly in the textile, cosmetic, food, and medical industries [2,3,4].

The biological activity of alginate oligosaccharides (AOS), comprised of alginate oligomers containing 2 to 25 monomers, has been recognized. AOS is exploited in food, medicine, and agriculture. AOS has antioxidant, anti-inflammatory, prebiotic, and antibacterial activities, among others, with the M/G ratio and molecular weight playing important roles in its potential bioactivity [5]. AOS with a low M/G ratio was reported to inhibit the pancreatic lipase [6]. AOS with a polymerization degree of 3–6 can induce cytokine synthesis [7], and AOS with a polymerization degree of 2–10 significantly inhibits the growth of human prostate cancer cells [8]. Furthermore, as a derivative of alginate oligosaccharides, propylene glycol alginate sodium sulfate (PSS) which is a heparinoid drug can prevent and treat hyperlipidemia and ischemic cardio-cerebrovascular diseases [9]. Oligomannuronic acid with two carboxyl groups at the reducing end (GV-971) reportedly improves cognition in patients with Alzheimer’s disease (AD) [10].

Alginate lyase can cleave alginate glycosidic bonds via a β-elimination mechanism, some of which have been isolated from algae, marine invertebrates and microorganisms [11,12]. Based on substrate specificity, alginate lyase can be classified into polyG lyases (EC 4.2.2.11), polyM lyases (EC 4.2.2.3), and polyMG lyases (EC 4.2.2.-). Due to the yield of alginate lyase in wild microorganism is low, heterologous expression is used to express alginate lyase. Some alginate lyases have been successfully expressed in *E. coli* [13,14,15,16]. We previously reported that an alginate lyase cAlyM with high enzymatic activity from *Microbulbifer* sp. Q7 (CGMCC 14061) expressed in *E. coli* [17]. However, *E. coli* is unable to secrete recombinant protein into the medium, and the application of the recombinant protein produced by *E. coli* is limited due to the safety of residual endotoxin.

Compared with *E. coli*, *P. pastoris* can secrete recombinant protein into the medium as soluble form, so it can be used to produce large quantities of enzymes by high-density fermentation without complicated purification steps [18]. *P. pastoris* expression system has significant advantages in the production of many recombinant proteins [19], but there are few reports of alginate lyases expressed in *P. pastoris* [20]. Moreover, due to the low activity and poor thermal stability of alginate lyases, their applications in the production of AOS have been limited [21]. Previous research on thermostable mutants of cAlyM in our laboratory has demonstrated that a thermostable mutant 102C300C, which has an additional disulfide bond expressed in *E. coli*, has good thermal stability, with the half-life value at 45 °C that is 2.18 times that of cAlyM [22].

To obtain a safe alginate lyase with high enzymatic activity and good thermal stability, the alginate lyase cAlyM and its thermostable mutant 102C300C were expressed in *P. pastoris*, and the enzymatic properties of these two enzymes were characterized and compared in detail. The degradation products were analyzed by high performance liquid chromatography (HPLC) and electrospray ionization mass spectrometry (ESI-MS).

## 2. Results and Discussion

### 2.1. Recombinant Expression of cAlyM and 102C300C

Due to the difference in codon usage bias between *P. pastoris* and wild microorganisms, the presence of rare codons in foreign genes reduced the expression level of recombinant proteins in the host. After optimization according to *P. pastoris* codon usage bias, foreign genes might reduce the diversity of tRNA and increase the expression level [18]. The non-optimized gene consisting of 951 bp from *Microbulbifer* sp. Q7 (CGMCC 14061) was optimized. The similarity between the non-optimized gene and optimized gene was 73.5%.

**Figure 1:**
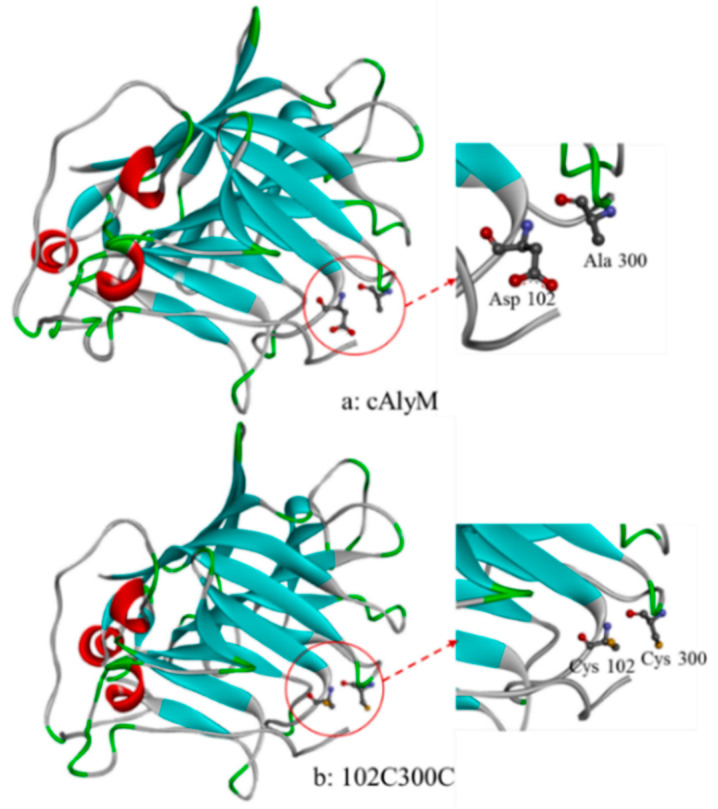
The position of mutation sites in the three-dimensional model of cAlyM (**a**) and 102C300C (**b**). The three-dimensional molecular visualization was performed using Phyre2. The arrows point to the magnified structures of the mutation sites.

In a previous study, the half-life value of the mutant 102C300C expressed in *E. coli* was 2.18 times that of cAlyM [22]. Therefore, to improve the thermal stability of the recombinant enzyme expressed in *P. pastoris*, an additional disulfide bond was introduced into cAlyM by site-directed mutagenesis. The aspartate acid (D) at position 102 and alanine (A) at position 300 were both mutated to cysteine (C). The positions of the mutation sites in the model are shown in Figure 1. The sites of mutation (102 and 300) were located on the surface of the enzyme, and were not inside the cavity where the enzyme bound to the substrate.

The recombinant plasmids, pPICZαA-cAlyM and pPICZαA-102C300C were successfully constructed and confirmed by DNA sequencing (Ruibiotech, Qingdao, China). These plasmids were transformed into *P. pastoris* X33 for protein expression. The recombinant yeast colonies, X33-cAlyM and X33-102C300C, fermented as induced by methanol in a shaking flask. The extracellular enzymatic activities of recombinant cAlyM and 102C300C were 33.82 U/mL and 33.87 U/mL, respectively. Based on the BLAST search (http://blast.ncbi.nlm.nih.gov/Blast.cgi), we determined that cAlyM and its thermostable mutant 102C300C belong to the PL-7 family.

### 2.2. Purification of Recombinant cAlyM and 102C300C

The cAlyM and 102C300C recombinant enzymes were purified using Ni^+^-chelated magnetic beads. After purification, the specific activities of cAlyM and 102C300C were 277.1 U/mg and 249.6 U/mg, respectively (Table 1). Due to the different definitions of enzyme activity units, the specific activities of many alginate lyases cannot be directly compared. For alginate lyases with the same definition of enzyme activity unit, the specific activities of cAlyM and 102C300C were higher than that of many reported alginate lyases. As two examples, the specific activity of the recombinant alginate lyase Aly7B_Wf expressed in *E. coli* from *Wenyingzhuangia fucanilytica* was 23.24 U/mg [23] and the specific activity of AlyH1 from *Vibrio furnissii* H1 was 2.40 U/mg [11]. The specific activity of cAylM expressed in *E. coli* was 1386.27 U/mg [17], which was higher than that expressed in *P. pastoris* and it might be caused by the glycosylation modification of enzyme. The predicted glycosylation sites in cAlyM are shown in Figure S1. The glycosylation sites were marked in the gene sequence and observed in the three-dimensional model of cAlyM (Figure S2). Some glycosylation sites were near the highly conserved regions, which might influence the accessibility of the substrate to the enzyme active site through steric hindrance and cause the decrease of the enzyme activity.

**Figure 2:**
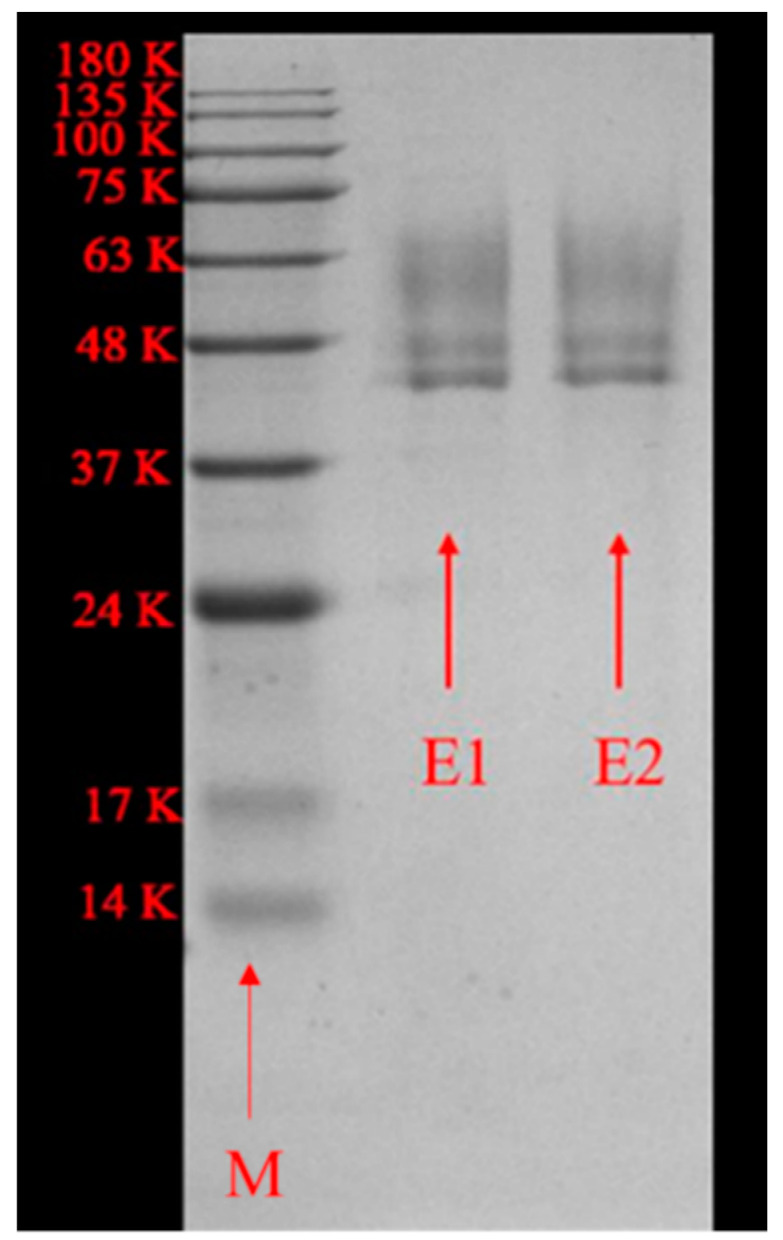
SDS-PAGE of recombinant cAlyM and 102C300C. Lane M, protein marker; lane E1, purified cAlyM; lane E2, purified 102C300C.

SDS-PAGE resolved two main protein bands with MWs of 43 kDa and 47 kDa in each sample (Figure 2). The calculated MW of cAlyM and 102C300C was 32.9 kDa. The difference between the actual value and the theoretical value may be explained by the different glycosylation modifications of the recombinant enzyme. Glycosylation of proteins during synthesis is an important modification that occurs during the secretion of proteins, resulting in the covalent linkage of carbohydrates to asparagine (N-linked) or to serine/threonine (O-linked) residues [24], and N-glycosylation affects the folding and transportation of proteins [25]. Various proteins have been cloned and expressed as glycoproteins in *P. pastoris.* The MW of glycosylated neutral protease reportedly increased from 43.3 kDa to 54.5 kDa [26] and the MW of glycosylated phytase increased from 54 kDa to 75 kDa [27].

### 2.3. Kinetic Parameters of cAlyM and 102C300C

The *K_m_* and *V_max_* values were calculated using the Lineweaver–Burk method. As shown in Table 2, cAlyM and 102C300C displayed similar *K_m_* values, which was lower than that of some reported alginate lyases [11,28]. For example, three alginate lyases derived from *Marinimicrobium* sp. H1 were expressed in *E. coli*, with *K_m_* values of 6.6, 6.9, and 7.7 mg/mL [13]. The *k_cat_* values of cAlyM and 102C300C were similar, which suggested that the mutations at position 102 and 300 did not cause structural changes in the catalytic activity center of the enzyme.

*K_m_* value indicated the affinity of enzyme and substrate. The *K_m_* values of cAlyM and 102C300C expressed in *E. coli* were 0.37 and 0.28 mg/mL, respectively [22], which were lower than those expressed in *P. pastoris*. The decrease of affinity of enzyme and substrate was related to the decrease of *V_max_* values, and the *V_max_* values of cAlyM and 102C300C expressed in *P. pastoris* were both lower than those expressed in *E. coli*.

### 2.4. Enzymatic Properties of cAlyM and 102C300C

**Figure 3:**
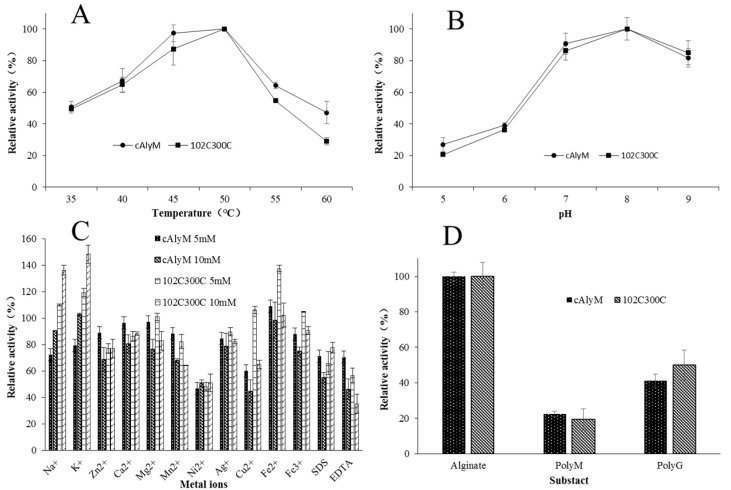
Biochemical properties of cAlyM and 102C300C. (**A**) The optimal temperatures of cAlyM and 102C300C. (**B**) Optimal pH of cAlyM and 102C300C. (**C**) Effect of metal ions on cAlyM and 102C300C. (**D**) Substrate preference of cAlyM and 102C300C. Values were reported as the mean of three determinations ± standard deviation.

The optimal temperature and pH, effects of metal ions, and substrate specificity of cAlyM and 102C300C were determined and compared. The enzymatic activities reached their optimum at 50 °C (Figure 3A). Many alginate lyases exhibit optimal enzymatic activity between 30 °C and 40 °C [21]. cAlyM and 102C300C displayed maximum activity at pH 8.0 (Figure 3B) and reached more than 80% activity at pH 7.0 and pH 9.0. These findings suggested that the enzymes can degrade alginate better under an alkaline condition. The slight changes in the surface charge of 102C300C did not cause the change in optimal pH of the enzyme.

Na^+^ and K^+^ (both 10 mM) enhanced 102C300C activity, while Ni^+^ and EDTA had obvious inhibitory effects on the enzymatic activity (Figure 3C). As the concentration of some ions such as Na^+^ and K^+^ increased, the relative activities of the two enzymes increased. As the concentration of Mg^2+^, Mn^2+^, and Fe^3+^ increased, the relative activities of the two enzymes decreased.

Substrate specificity analysis revealed activities toward sodium alginate, polyG, and polyM (Figure 3D), indicating that the enzymes were a bifunctional alginate lyase. The relative activities towards alginate and polyG were higher than that towards polyM. Some studies have reported that the substrate specificity of alginate lyase is related to the conserved amino acids in the gene [29]. The mutation sites of 102C300C were located on the surface of the protein and were not conserved amino acids, and so did not cause a significant change in substrate specificity.

### 2.5. Thermal Stability of cAlyM and 102C300C

**Figure 4:**
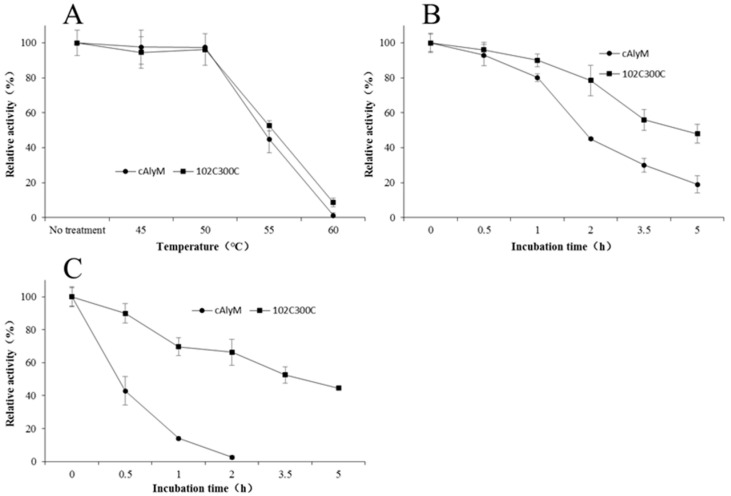
Thermal stability properties of cAlyM and 102C300C (**A**) Residual activities of cAlyM and 102C300C after incubation at different temperatures for 5 min. Residual activities of cAlyM and 102C300C after incubation at 45 °C (**B**) and 50 °C (**C**) for different times. Values were reported as the mean of three determinations ± standard deviation.

Mutant 102C300C was designed for the thermal stability of cAlyM, so the difference in thermostability between the two enzymes was important to determine, to judge whether the mutation was favorable. After incubation at 45 °C, 50 °C, 55 °C, and 60 °C for 5 min, the difference in relative activity was evident at temperatures of 50°C and above. The remaining enzymatic activities of 102C300C at 55 °C and 60 °C were higher than those of cAlyM (Figure 4A). After treatment at 55 °C for 5 min, the relative activities of cAlyM and 102C300C were 44% and 52% of the original activity, respectively. The half-life values at 45 °C (t_1/2,45°C_) of cAlyM and 102C300C were 2.0 h and 5.2 h, respectively, and the t_1/2,45°C_ of the mutant were 2.6 times higher than that of cAlyM (Figure 4B,C). The half-life values at 50 °C (t_1/2,50°C_) of cAlyM and 102C300C were 0.3 h and 3.5 h, respectively, and the t_1/2,50°C_ were 11.7 times higher than that of cAlyM. The results indicated that the thermal stability of 102C300C had improved. The data indicated that the strategy of adding disulfide bonds within the protein to improve protein thermal stability is feasible in the rational design of enzymes.

Compared with many reported alginate lyases, 102C300C exhibited good thermostability (Table 3). For example, the recombinant alginate lyase algA expressed in *E. coli* from *Pseudomonas* sp. E03 lost 50% activity at 50 °C for 30 min [28]. KJ-2 expressed in *E. coli* from *Stenotrophomas maltophilia* was inactivated at temperatures exceeding 40 °C for 30 min [30]. The relatively higher specific activity and good thermostability of 102C300C favor its use in the production of AOS. Among the reported alginate lyases, some alginate lyases showed a good thermostability. SAGL expressed in *P. pastoris* from *Flavobacterium* sp. H63 retained 49.0% activity at 50 °C for 72 h, which had polyM preference [20]. AlgC-PL7 expressed in *E. coli* from *Cobetia* sp. NAP1 retained 80% activity at 70 °C for 1 h [31]. GLyase from *Pseudomonas* sp. F6 retained 60% activity at 80 °C for 15 min [32].

### 2.6. ESI-MS and HPLC Analyses of Degradation Products

**Figure 5:**
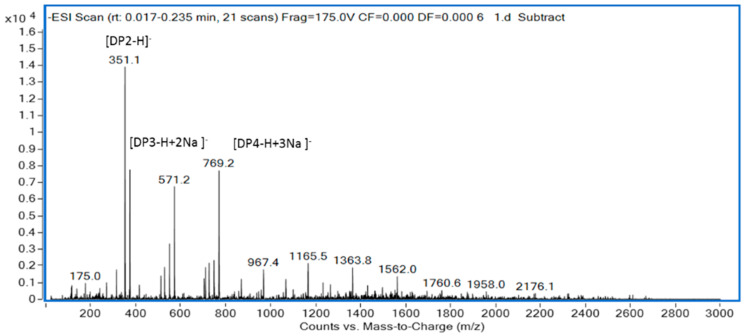
ESI-MS of the102C300C degradation products.

The degradation products of 102C300C were identified by ESI-MS. Ions at 351, 571, and 769 m/z represented unsaturated disaccharides (352 Da), unsaturated trisaccharides (572 Da), and unsaturated tetrasaccharides (770 Da), respectively (Figure 5). It proved that oligosaccharides with a polymerization degree of 2–4 were the final hydrolytic products. Compared with the analysis of the degradation products of cAlyM [34], the ESI-MS result of 102C300C was consistent with that of cAlyM, indicating that the mutant did not change the action model of alginate lyase.

HPLC analysis revealed that the degradation products precipitated by 1-, 3-, and 5-fold ethanol had MWs of 4.92 kDa, 3.49 kDa, and 2.31 kDa, respectively (data not shown). The yields of the degradation products precipitated by 1-, 3-, and 5-fold ethanol were 11.67%, 83.55% and 2.20%, respectively, and the yield of unhydrolyzed part was 0.98%. As shown in Table 4, the M/G ratios of the degradation products precipitated by 1-, 3-, and 5-fold ethanol were 2.44, 0.85, and 0.37, respectively. As the MW of the degradation product decreased, the proportion of G in the monosaccharide composition gradually increased, indicating a preferential polyG substrate specificity of 102C300C, which was consistent with the result of substrate specificity. Many reports have described immunoregulatory, antimicrobial, and anti-inflammatory activities of α-L-guluronic acid oligosaccharides (GOS), the degradation product of alginate. For example, GOS displays anti-inflammatory activity on lipopolysaccharide-activated murine macrophages [35]. GOS can strengthen the action of antibiotics and destroy bacterial biofilms by binding to the bacterial surfaces, regulating surface charges, inducing microbial aggregation, and inhibiting motility [36].

## 3. Materials and Methods

### 3.1. Strains, Plasmids, and Reagents

The alginate lyase gene cAlyM from *Microbulbifer* sp. Q7 (CGMCC 14061) was optimized according to *P. pastoris* codon usage bias. The codon-optimized gene synthesized by Shanghai TY Biotechnology Co. Ltd. (Shanghai, China) was inserted into the plasmid pPIC9K. The recombinant plasmid was named as pPIC9K-cAlyM. *E. coli* DH5α, *P. pastoris* strain X33, and plasmid pPICZα were conserved in our laboratory. *E. coli* DH5α was used for plasmid construction. *P. pastoris* X33 was used for eukaryotic expression. Sodium alginate (M/G ratio 0.85), polyM, and polyG blocks (purity > 90%) were purchased from Qingdao BZLH Biotech Co. Ltd. (Qingdao, China). The antibiotic zeocin was purchased from Beijing Solaribio Technology Co. Ltd. (Beijing, China). Restriction enzymes *EcoR*I, *Not*I, and *Sac*I were purchased from Thermo Fisher Scientific (Waltham, MA, USA).

### 3.2. Construction of Alginate Lyase and Mutant Recombinant Plasmid

The codon-optimized alginate lyase gene was amplified from pPIC9K-cAlyM by PCR using 5′-AGAGAGGCTGAAGCTGAATTCACTGAATCTGGTTCTGGTTCTTCTT-3′ as the upstream primer and 5′-TGTTCTAGAAAGCTGGCGGCCGCTTAGTGGTGATGGTGATGATGATC-3′ as the downstream primer. Plasmid pPICZα was digested with *EcoR*I and *Not*I. The purified cAlyM fragment was ligated into plasmid pPICZα at the *EcoR*I and *Not*I sites. The recombinant plasmid was named as pPICZα-cAlyM.

Site-directed mutagenesis was used to construct the mutant recombinant plasmid. The recombinant plasmid pPICZα-cAlyM was used as the template to amplify the mutation plasmids by PCR using the following primers: D102C-F: ATGTCCAATCTGTGGTTACAAGACTTCTACCAACACCTCC; D102C-R, TCTTGTAACCACAGATTGGACATCTGAACACCATACCAC, A300C-F: TACTGGTAACTGTTCCGACTACGTTCAGGTTACTTTCTAC, and A300C-R: CGTAGTCGGAACAGTTACCAGTATTGTTCTGGTTGTAAACAC. *Dpn*I was used to remove the template DNA from the PCR product. The linearized PCR product was purified and ligated. The mutant recombinant plasmid was named as pPICZα-102C300C.

### 3.3. Transformation and Colony Screening of P. pastoris

Recombinant plasmids pPICZα-cAlyM and pPICZα-102C300C were digested with *Sac*I. The linearized form of the recombinant plasmids were transformed into *P. pastoris* X33 competent cells by electroporation at 2.0 kV using a one-pulse electroporation cuvette, and selected on YPDS plates containing 100 μg/mL zeocin. After 72 h, 24 yeast colonies were picked from each plate and cultivated in 1 mL BMGY by inoculating into a 48-pore plate at 30 °C and 200 rpm for 72 h. Methanol was added to a final concentration of 1% every 24 h to induce the expression of alginate lyase. After the enzymatic activity assay, some positive recombinant yeast colonies were inoculated in 20 mL YPD medium at 30 °C and 200 rpm for 24 h. The cells were collected by centrifugation 10,000 rpm at 4 °C for 10 min, resuspended in 20 mL BMGY medium, and incubated at 30 °C and 200 rpm for 72 h with methanol added to a final concentration of 1% every 24 h. Positive recombinant yeast colonies were named as X33-cAlyM and X33-102C300C, respectively.

### 3.4. Purification of Recombinant cAlyM and 102C300C

The recombinant enzymes cAlyM and 102C300C fused with a His6 tag were purified using Ni^+^-chelated magnetic beads (Suzhou Beaver Biomedical Co. Ltd., Suzhou, China). The manufacturer’s purification protocol was followed. The concentrations of imidazole in the binding buffer and elution buffer were 50 mM and 100 mM, respectively. The purified enzymes were further analyzed by 12% SDS-PAGE. The protein concentration was determined by the Coomassie Brilliant Blue method and measured by absorbance at 295 nm [37]. The purified recombinant enzymes were named as cAlyM and 102C300C.

### 3.5. Enzymatic Activity Assay

Enzyme activity was determined by the 3,5-dinitrosalicylic acid (DNS) method [38]. Nine hundred microliters of sodium alginate (0.8%, pH 7.0) was mixed with 100 μL of the recombinant enzyme and reacted at 45 °C for 5 min. The reaction was terminated by adding 1 mL of DNS solution. The mixture was incubated in a boiling water bath for 5 min and ddH_2_O was added to a total volume of 10 mL. Absorbance was measured at 540 nm. One unit of the enzyme (U) was defined as the amount of enzyme causing the release of 1 μmol of reducing sugar from alginate per minute.

### 3.6. Kinetic parameters of cAlyM and 102C300C

The kinetic parameters of the enzymes were determined by measuring the enzyme activities of the purified enzymes at different concentrations (0.5–8 mg/mL) of sodium alginate. The *K_m_* and *V_max_* values were calculated using the Lineweaver–Burk method.

### 3.7. Enzymatic Properties of cAlyM and 102C300C

The optimal catalytic temperatures of cAlyM and 102C300C were determined by measuring activity from 35 °C to 60 °C in 100 mM phosphate buffer (pH 7.0). The optimal catalytic pH value was determined by measuring activity at various pH values ranging from 5.0 to 9.0 in 100 mM phosphate buffer. Substrate specificity was determined by measuring activity upon reaction with 2 mg/mL of sodium alginate, polyM, and polyG. The highest enzyme activity was taken as 100%.

To determine the influence of metal ions on the activity of cAlyM and 102C300C, the recombinant enzymes were incubated with various metal ions at a final concentration of 5 mM and 10 mM at 4 °C for 2 h. Activity was measured and the reaction mixture without any metal ions was taken as 100%. The metal ions included Na^+^, K^+^, Zn^2+^, Ca^2+^, Mg^2+^, Mn^2+^, Ni^2+^, Ag^+^, Cu^2+^, Fe^2+^, Fe^3+^ and SDS.

### 3.8. Thermal Stability of cAlyM and 102C300C

The recombinant enzymes were incubated at various temperatures ranging from 45 °C to 60 °C for 5 min, and the residual enzyme activity of the sample was measured. To determine the half-life value, the residual activity was measured after incubating at 45 °C and 50 °C for different times. The reaction mixture without any heat treatment was taken as 100%.

### 3.9. ESI-MS and HPLC Analyses of Degradation Products

The sample prepared through the hydrolysis of 102C300C on alginate was analyzed by ESI-MS and HPLC. In this hydrolysis reaction, 2% sodium alginate was degraded at 45 °C for 8 h by adding 1% 102C300C every 2 h. The degradation products were vacuum freeze-dried and analyzed by negative ion ESI-MS (Agilent 1290 Infinity II-6460, Frag = 175.0 V, m/z 100–2000 amu).

To determine the molecular weight (MW) of the degradation products, the degradation products were prepared by gradient ethanol precipitation. The precipitates were freeze-dried and analyzed by HPLC (Agilent 1260 Infinity HPLC system) using a PL Aquagel-OH 30 column (Agilent, Santa Clara, CA, USA). NaNO_3_ (200 mM) with 10 mM NaH_2_PO_4_ was used as the mobile phase at a flow rate of 0.5 mL/min. The column temperature was 25 °C and a refractive index detector (RID) was used. Dextrans (MWs: 1, 3.65, 5 and 12 kDa) were used as standards. Before injection, the precipitates were dissolved in the mobile phase and passed through a 0.22 μm filter. To determine the monosaccharide composition of the degradation products, the precipitates were decomposed by 2 M trifluoroacetic acid (TFA) at 110 °C for 4 h and detected by HPLC using an XDB-C18 column (Agilent) after pre-column derivatization using 1-phenyl-3-methyl-5-pyrazolone (PMP). KH_2_PO_4_ (50 mM, pH 6.9) was used as the mobile phase at a flow rate of 1 mL/min. The column temperature was 25 °C and detection was done using an ultraviolet detector at 245 nm. The standards were mannuronate monosaccharide and guluronate monosaccharide.

## 4. Conclusions

With codon optimization of the alginate lyase gene, the recombinant alginate lyase cAlyM and its thermostable mutant 102C300C were expressed and characterized in *P. pastoris*. 102C300C displayed higher activity and thermal stability than previously reported alginate lyases. The specific activity of 102C300C was 249.6 U/mg and its t_1/2,45°C_ was 5.2 h. Since alginate lyases are widely applied for the preparation of functional oligosaccharides, seaweed fertilizers and feed additives, which areusually performed under high temperature. This work provides a valuable reformation method for thermostable alginate lyases to meet the requirement of industrial application.

## Figures and Tables

**Table 1:** Purification of cAlyM and 102C300C.

Enzymes	Step	Total Activity (U)	Total Protein (mg)	Specific Activity (U/mg)	Recovery Rate (%)	Fold	Yield (U/mL)
cAlyM	Fermentation medium	507.4 ± 8.5	3.00 ± 0.06	169.1	100	1	33.8 ± 0.57
	Magnetic beads	188.4 ± 4.6	0.68 ± 0.01	277.1	37.13	1.64	26.9 ± 0.66
102C300C	Fermentation medium	617.6 ± 6.4	3.82 ± 0.05	161.7	100	1	41.2 ± 0.43
	Magnetic beads	237.1 ± 5.1	0.95 ± 0.01	249.6	38.39	1.54	33.9 ± 0.73

**Table 2:** Enzyme kinetic parameters of cAlyM and 102C300C.

Enzyme	*V_max_* (U/mg)	*K_m_* (mg/mL)	*k_cat_* (s^−1^)	*k_cat_*/*K_m_* (mL/s/mg)
cAlyM	344.8 ± 5.6	1.31 ± 0.21	189.1 ± 5.3	144.3
102C300C	312.5 ± 10.1	1.43 ± 0.20	171.3 ± 4.3	119.2

**Table 3:** Data of some reported alginate lyases.

Enzyme	Origin	Source	Substrate Preference	Optimal Catalytic Temperature	Thermalstability	Specific activity (U/mg)	Reference
Aly08	*Vibrio* sp. SY01	*E. coli*	Poly G	45 °C	Retained 48.7% activity at 20 °C for 1 h	841	[14]
Aly7B_Wf	*Wenyingzhuangia fucanilytica*	*E. coli*	Poly M	40 °C	Retained 75% activity at 35 °C for 24 h	23.24 *	[23]
AlyH1	*Vibrio furnissii* H1	Native	Poly G	40 °C	Retained 60% activity at 40 °C for 30 min	2.40 *	[11]
FsAlgB	*Flammeovirga* sp. NJ-04	*E. coli*	Poly M and alginate	40 °C	Retained 80% activity at 40 °C for 30 min	1760.8	[15]
AlgH	*Marinimicrobium* sp. H1	*E. coli*	Poly G	45 °C	Retained 80% activity at 40 °C for 2 h	5510	[13]
PmC5A	*Pseudomonas mendocina* DICP-70	*E. coli*	Poly G and Poly M	40 °C	Retained 80% activity at 45 °C for 1 h	N.D.	[16]
Aly1281	*Pseudoalteromonas carrageenovora* ASY5	*E. coli*	Poly G	50 °C	Stable at temperatures lower than 55 °C	1.15 *	[33]
Alg17B	BP-2	Native	Poly M	45 °C	Retained 10% activity at 45 °C for 1 h	4036	[12]
KJ-2	*Stenotrophomas maltophilia* KJ-2	*E. coli*	Poly MG	40 °C	Inactivated at higher than 40 °C for 30 min	848.3	[30]
algA	*Pseudomonas* sp. E03	*E. coli*	Poly M	30 °C	Retained 50% activity at 50 °C for 30 min	222	[28]
SAGL	*Flavobacterium* sp. H63	*P. pastoris*	Poly M and alginate	45 °C	Retained 49.0% activity at 50 °C for 72 h	4044 *	[20]
AlgC-PL7	*Cobetia* sp. NAP1	*E. coli*	Poly G and Poly M	45 °C	Retained 80% activity at 70 °C for 1 h	30	[31]
GLyase	*Pseudomonas* sp. F6	Native	Poly G	N.D.	Retained 60% activity at 80 °C for 15 min	222.8	[32]
cAlyM	*Microbulbifer* sp. Q7	*P. pastoris*	Poly G and alginate	50 °C	Retained 50% activity at 45 °C for 2 h	277.1 *	This study
102C300C	*Microbulbifer* sp. Q7	*P. pastoris*	Poly G and alginate	50 °C	Retained 50% activity at 45 °C for 5.2 h	249.6 *	This study

Note: “*” indicates that alginate lyase activity is measured using the DNS method. Alginate lyase activity not denoted with “*” was measured by the 235 nm absorbance method and one unit of activity was defined as the amount of enzyme required to increase the absorbance at 235 nm by 0.1 per min.

**Table 4:** M/G ratios and yield of degradation products precipitated using different volumes of ethanol.

Sample	Peak Area Percentage of G	Peak Area Percentage of M	M/G
1-fold	15.05%	84.95%	5.61
3-fold	31.17%	68.83%	2.08
5-fold	72.91%	27.09%	0.37

## Supplementary Materials

The following are available online at https://www.mdpi.com/1660-3397/18/6/305/s1. Figure S1: The prediction results of O-glycosylation (A) and N-glycosylation (B) of cAlyM. Figure S2: The predicted glycosylation sites in the gene sequence of cAlyM (A). The predicted N-glycosylation sites in the three-dimensional model of cAlyM (B).
