# Supplementary material for: Expression and Characterization of an Alginate Lyase and Its Thermostable Mutant in Pichia pastoris
Source: Mar Drugs. 2020 Jun 11;18(6):305. doi: 10.3390/md18060305 (PMC7345639; doi:10.3390/md18060305)
Supplement: Supplementary file 1 [file marinedrugs-18-00305-s001.pdf]

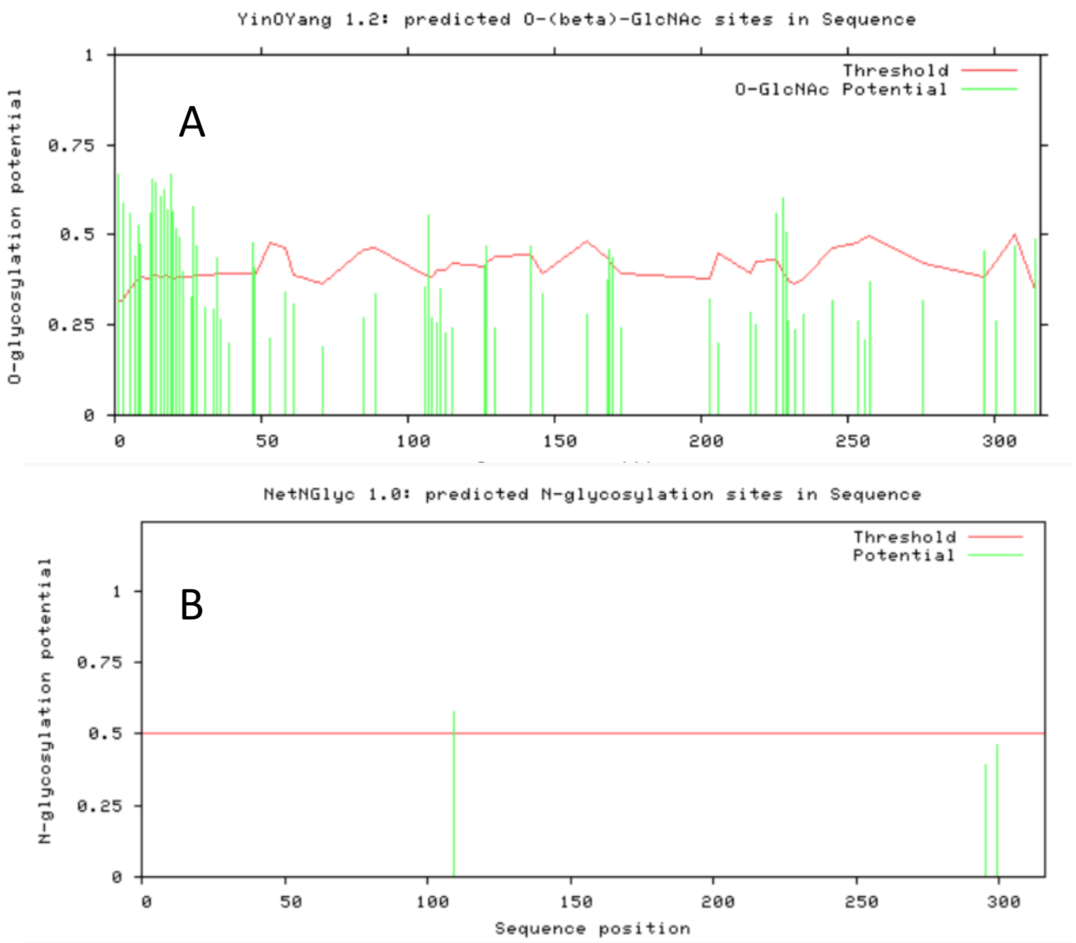

Figure S1. The prediction results of O-glycosylation (A) and N-glycosylation (B) of cAlyM. The glycosylation sites were predicted using the website (http://www.cbs.dtu.dk/services/NetNGlyc/, http://www.cbs.dtu.dk/services/YinOYang/).

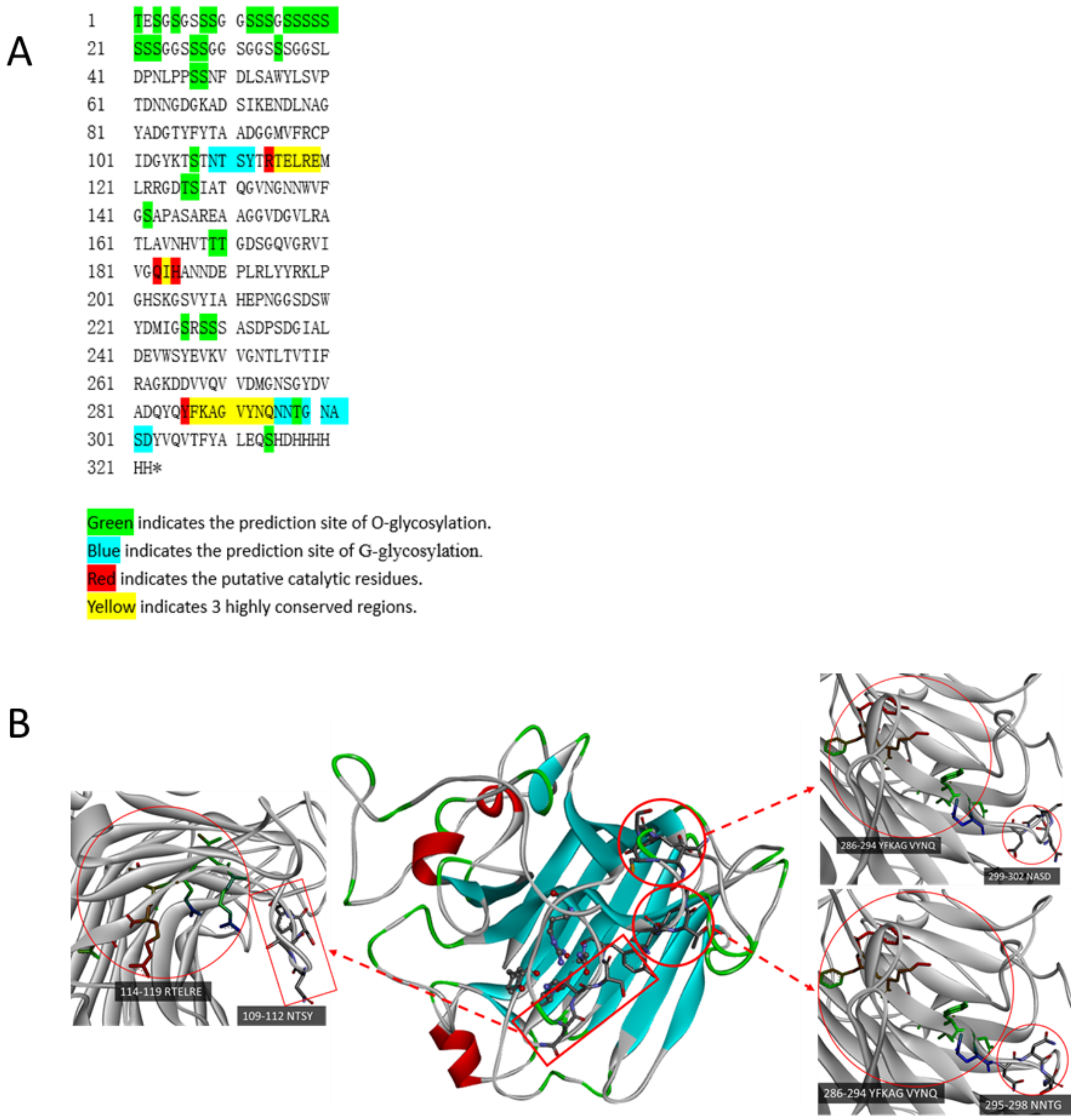

Figure S2. The predicted glycosylation sites in the gene sequence of cAlyM (A). The predicted N-glycosylation sites in the three-dimensional model of cAlyM (B). A: Green indicates the prediction site of O-glycosylation. Blue indicates the prediction site of G-glycosylation. Red indicates the putative catalytic residues. Yellow indicates 3 highly conserved regions. B: The arrows point to the magnified structures of the mutation sites.
